# Consequences of Lower Food Intake on the Digestive Enzymes Activities, the Energy Reserves and the Reproductive Outcome in *Gammarus fossarum*

**DOI:** 10.1371/journal.pone.0125154

**Published:** 2015-04-16

**Authors:** Laetitia Charron, Olivier Geffard, Arnaud Chaumot, Romain Coulaud, Ali Jaffal, Véronique Gaillet, Odile Dedourge-Geffard, Alain Geffard

**Affiliations:** 1 Université Reims Champagne Ardenne, Unité Mixte de Recherche-Ineris (UMR-I02) Stress Environnementaux et Biosurveillance des milieux aquatiques, Unité de Formation et de Recherche Sciences Exactes et Naturelles, Moulin de la Housse, Reims Cedex 2, France; 2 Institut national de recherche en sciences et technologies pour l’environnement et l’agriculture, Unité de Recherche Milieux Aquatiques, Ecologie et Pollutions, Ecotoxicologie, Villeurbanne Cedex, France; Sonoma State University, UNITED STATES

## Abstract

Digestive enzyme activity is often used as a sensitive response to environmental pollution. However, only little is known about the negative effects of stress on digestive capacities and their consequences on energy reserves and reproduction, although these parameters are important for the maintenance of populations. To highlight if changes in biochemical responses (digestive enzymes and reserves) led to impairments at an individual level (fertility), *Gammarus fossarum* were submitted to a lower food intake throughout a complete female reproductive cycle (i.e. from ovogenesis to offspring production). For both males and females, amylase activity was inhibited by the diet stress, whereas trypsin activity was not influenced. These results underline similar sensitivity of males and females concerning their digestive capacity. Energy reserves decreased with food starvation in females, and remained stable in males. The number of embryos per female decreased with food starvation. Lower digestive activity in males and females therefore appears as an early response. These results underline the ecological relevance of digestive markers, as they make it possible to anticipate upcoming consequences on reproduction in females, a key biological variable for population dynamics.

## Introduction

The environmental risk of aquatic ecosystems is continuously monitored by the application of chemical and biological tools. In this way, many biological responses to stress have been investigated as diagnosis tools. These measurements are well-known biomarkers used to assess the effects of toxic exposure on organisms. According to Depledge and Fossi [1], biomarkers are defined as “biochemical, cellular, physiological or behavioral variations that can be measured in tissue or body fluid samples or at the level of whole organisms that provide evidence of exposure to and/or effects of, one or more chemical pollutants (and/or radiations)”. The interest of these biological tools to link contaminant exposure with animal health impairment has been clearly demonstrated [2]. However, the significance of such sub-organismal responses in relation to impacts at higher levels of biological organization (population and/or ecosystem) is uncertain and often poorly understood [3]. In fact, there is a knowledge gap about the ecological relevance of most of the currently implemented biomarkers [4]. Therefore, validation studies demonstrating quantitative relationships between sub-organismal and supra-organismal effects need to be developed [5–7].

Among all the biological responses studied in ecotoxicology, energy-based biomarkers seem to be a relevant tool for investigating the relationships between disturbances at the sub-organismal level and disturbances emerging at higher levels of biological organization [8, 9]. According to the hypothesis proposed by De Coen and Janssen [8, 10], sublethal stress induces changes in the energy budget. To cope with stress, energy expenditure for basal metabolism increases. In parallel, the energy available for growth and reproduction is reduced. So energy analysis can (i) reveal physiological disturbances in organisms and (ii) make the link with key biological variables for population dynamics such as survival, growth and reproduction. Many different energy parameters have been already used as contamination biomarkers, such as the adenylate energy charge (AEC) [11], energy reserves (glycogen, lipids, proteins) [12], cellular energy allocation (CEA) [13] and the scope for growth (SfG) [14].

Furthermore, to assess the global energy metabolism, previous processes concerning energy acquisition should be considered. Energy intake depends on feeding and digestion processes. It could affect the energy metabolism and lead to its impairment. In aquatic invertebrates, feeding rates and digestive enzyme activity are usually studied as sensitive responses to environmental pollution [15–20]. Many studies have shown the negative effect of chemical contamination of waters on ingestion and assimilation processes in aquatic organisms. Nevertheless, very few works have considered the effect of feeding and digestive inhibition on the energy metabolism and its consequences on the reproductive success at an individual level. However, energy metabolism is directly linked with key biological variables such as growth or reproduction [6, 21].

In European freshwater ecosystems, amphipods are usually considered as keystone species due to their high density and their position in the aquatic food web. The *Gammarus* genus is a shredder and a detritus feeder that plays a major role in the processing of organic matter [22] and is prey to many other organisms [23]. Therefore, the structure and functioning of freshwater ecosystems are strongly influenced by gammarid populations, underlining their high ecological relevance [24]. Moreover *Gammarus sp*. are currently used in aquatic ecotoxicological studies as sentinel species owing to their sensitivity to several toxicants. The reproductive cycle of *Gammarus sp*. is well described [25], and the relationships between responses at different biological levels have been studied for specific markers (neurotoxicity: acetylcholinesterase activity; genotoxicity: comet assay) [5, 7]. In freshwater gammarids, some studies show that inhibition of the feeding behavior and/or lower digestive activity can be caused by a wide range of pollutants (metals, pesticides, organic pollutants) [19, 26]. Finally, gammarids are also relevant test species because of their relatively short, temperature-dependent, reproductive cycle [27], with several broods *per* year [28]. Thus, the whole ecological and physiological features of gammarids enable investigations on early changes during energy acquisition as well as long-term effects on their reproductive success [24, 29]. The interest of digestive enzymes as potential biomarkers in *G*. *fossarum* has been underlined in previous studies [26]. Moreover, the optimal conditions for their assay as biomarkers have been defined, including the use of males (to avoid the strong influence of the female reproductive cycle [30]), under an active approach to be able to feed them *ad libitum*. However, in these conditions, clarifying the precocious character of digestive enzyme assays according to responses at the individual level (growth, reproduction) appears necessary to determine their ecological relevance as biomarkers at the species level.

In order to assess how monitoring digestive activity and energetic marker levels could be used to anticipate adverse outcomes on reproductive capacities, *G*. *fossarum* were submitted to stress throughout a complete female reproductive cycle (i.e. from ovogenesis to offspring production). As males were recommended to use digestive enzyme as biomarkers, the present study was based on the two genders in view to compare their patterns of responses and to define if the measurement in males could be translated in females. Amylase and trypsin activity levels, energy reserves and fertility (number of embryos) were measured at different times. Nutritional stress was used as a means to assess the relationship between energy intake disturbances and the reproductive outcome, regardless of the impacts of chemical stress.

## Materials and Methods

### Ethics statement

Gammarids were sampled by members of the Institut national de recherche en sciences et technologies pour l’environnement et l’agriculture (Irstea), which is a French national institute of science and technology for environment and agriculture.

In France, amphipod research does not require permission, *G*. *fossarum* is not a protected species and its use in scientific research does not require any specific authorization. Gammarids were sampled at “La Tour du Pin” (E: 5°27'33" N: 45°34'10") and no specific permissions were required for this location. All efforts were made to minimize suffering during laboratory experiments.

### Sampling and maintenance of gammarids

Male and female *Gammarus fossarum* were collected with a hand-held net (2–2.5mm) at La Tour du Pin, upstream of the Bourbre River (France) [19, 31]. The station has good water quality according the data records of the RNB (French Watershed Biomonitoring Network). After sampling, the gammarids were brought to the laboratory, where they were kept in 30-L tanks under constant aeration at 14±0.5°C using a 10/14-hr light/dark photoperiod. The tanks were continuously supplied with drilled groundwater mixed with softwater (obtained by reverse osmosis) to adjust to the sampling site conductivity of 600 μS/cm. Throughout the 15 days of the acclimatization period, gammarids were fed *ad libitum* with alder leaves (*Alnus glutinosa*) collected from a pristine site and previously conditioned for at least 6 (±1) days in groundwater. Freeze-dried *Tubifex sp*. worms were added as a dietary supplement twice a week.

### Experimental design: food starvation experiment

The aim of our study was (1) to define the temporal dynamics (i.e. sensitivity) of digestive enzyme activity and energetic marker responses in case of feeding inhibition, and (2) to determine if monitoring digestive activity and energetic marker levels could be used to anticipate adverse outcomes on the reproductive capacities of female *G*. *fossarum*. According to Geffard and collaborators [25], the length of a female reproductive cycle is 30 days in these conditions (14°C, fed *ad libitum*).

Diet starvation was applied throughout two successive reproductive cycles by exposing concomitantly 70 pairs of gammarids (70 males and 70 females) in each feeding regime. The 70 females were exposed just after they molted (thus initiating a new reproductive cycle). The experiment was carried on over a second cycle (43 days after the start of the experiment, i.e. of the first reproductive cycle) to measure effective reproduction (fertility) under the different starvation conditions. At that time point, marsupium embryos were issued from the fertilization of oocytes developed during the first cycle. According to previous experiments [25], starvation conditions of 0% (control), 50% or 75% were gradually applied. In the control condition, gammarids were fed *ad libitum* every day of the week with 2-cm diameter alder leaf discs (*Alnus glutinosa*) (control condition, noted 7/7). In the other two conditions, gammarids were fed by supplying alder leaf discs only for 2 consecutive days a week (Monday and Tuesday, 50% food starvation, noted 2/7) or only 1 day a week (Monday, 75% food starvation, noted 1/7). After each feeding period for the 2/7 and 1/7 conditions, the remaining food was removed from the beakers and kept in a cold room for the next meal.

**Fig 1:**
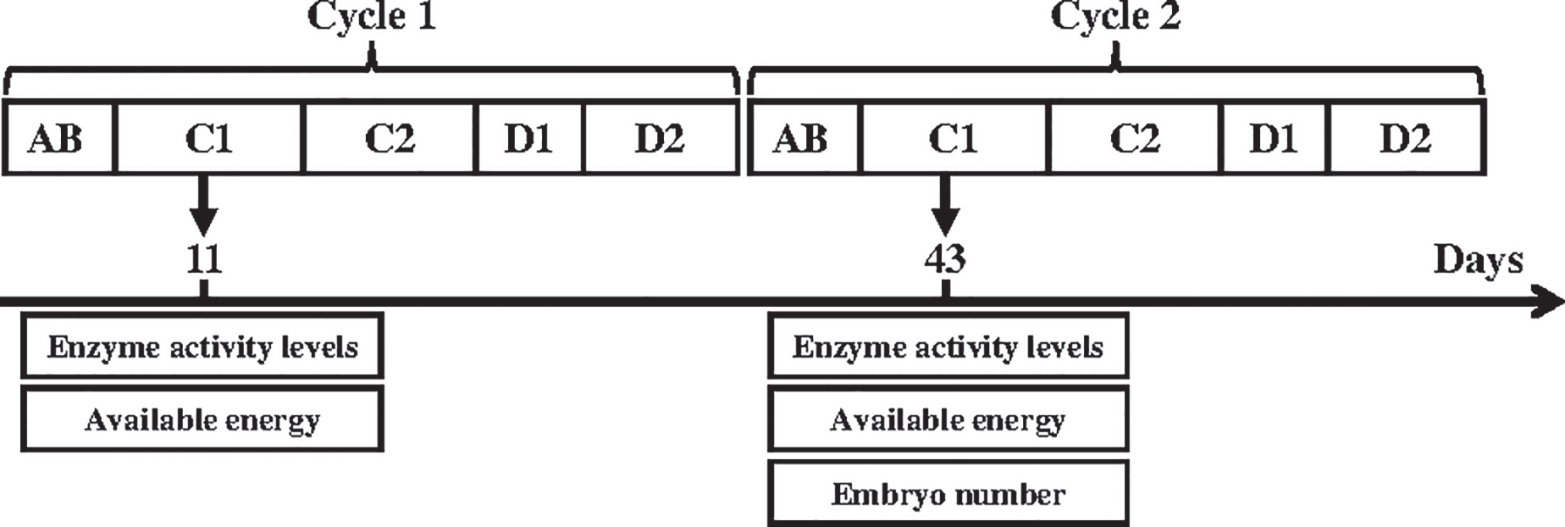
Female reproductive cycle stages (AB, C1, C2, D1 and D2, according to Geffard et al. [25]) and sampling periods for biological response measurements in males and females during the food starvation experiment.

Female and male gammarids were sampled after 11 days during the first reproductive cycle to assess energy parameters, and after 43 days during the second reproductive cycle to assess energy and fertility parameters (Fig 1). In accordance with the reproductive cycle in the control conditions at 14±0.5°C [25], females were expected to be in the C1 (11 and 43 days) stage of their reproductive cycle. The molting stages of females were assessed throughout the experiment, and only females in AB or C1 stage were considered for biochemical analysis and fertility measurements, to avoid potential effects of a delay in the molting cycle. To accurately assess the female molt stages, the third and fourth periopod pairs (dactilopodite and protopodite) of females were cut off, mounted on a microscope slide and covered with a coverslip, and their integumental morphogenesis was observed (x 200) to discriminate among the five molt stages (AB, C1, C2, D1 and D2). Oocyte maturation and embryo development in the marsupium take place simultaneously in female gammarids in the course of the molt cycle.

For each exposure condition, (11 and 43 days; food starvation levels: 0, 50 and 75%), 6 gammarids (males and females separately) were sampled for energy reserves and 6 pools of 3 gammarids (males and females separately) were collected for digestive enzyme activity assays. In all cases, embryos were collected manually from the marsupium and eliminated before biochemical analysis. Therefore, during the second cycle (after 43 days), the number of embryos *per* female (issued from the first cycle) was determined by collecting embryos from the marsupium and counting them [25] on the females used for energy reserve measurements. All samples were weighed, frozen in liquid nitrogen and stored at -80°C until analysis.

### Measurement of digestive enzyme activity levels and energy reserves in *G*. *fossarum*

#### Digestive enzyme activity

Amylase and trypsin activity levels were measured according to modified methods from Palais *et al*. [32] and Garcia-Carreño and Haard [33], respectively, with starch (1%) and BAPNA (3mM) as substrates. Calibration curves were established with maltose and p-nitroaniline (p-Na) for amylase activity and trypsin activity, respectively.

Each enzyme activity level was expressed as micrograms of the final product released *per* minute and *per* milligram of protein. Total and cytosolic protein contents in the supernatant were determined according to Bradford [34], using bovine serum albumin (BSA) as a protein standard.

#### Energy reserves

A method adapted from Van Handel [35, 36] and Plaistow *et al*. [37] was used to measure lipid, free sugar and glycogen contents in single individuals. Each gammarid was homogenized in 800μL of methanol containing 3 stainless steel balls by using a Mixer Mill MM400 (Retsch, Haan, Germany). Homogenization was performed for 2 min at 30 Hz. The homogenate was divided into two identical volumes (A and B) to measure lipids and sugars (free sugars and glycogen).

To measure total lipids in homogenate (A), 200μL of chloroform were added and mixed. After 20 min at 4°C, 100μL of homogenate were transferred into culture tubes and placed in a water bath at 95°C to evaporate the solvent. Then, 200μL of sulfuric acid (95%) were added to the mixture and left at 95°C for 10 min. Finally, the tubes were placed in an ice water bath and 5 ml of vanillin-phosphoric acid reagent were added. After 25 min, the optical density was read at 525 nm. Olive oil (Sigma Aldrich) solubilized in chloroform (1g/L) was used as a standard.

To measure sugars in homogenate (B), 200μL of sodium sulphate (2%) were added and mixed. After 20 min at 4°C, the samples were centrifuged (2,000xg, 4 min, 4°C). The resulting supernatant (solution 1) and the pellet were respectively used to measure free sugar and glycogen contents. Three hundred μL of supernatant (solution1) were transferred into culture tubes, while each pellet was resuspended in 400μL of distilled water (solution 2) with three stainless steel balls, and shaken for 1 min at 30Hz. Subsequently, 300μL (solution 2) of glycogen homogenate were placed in culture tubes. Finally, 5 mL of anthrone reagent were mixed into every sample (solutions 1 and 2), and the tubes were heated at 95°C in a water bath for 17 min. The samples were cooled for 10 min, and their optical density was measured at 630 nm. Glucose solution (1g/L) was used as a standard.

Available energy (Ea) was deduced from the total protein, carbohydrate and lipid contents at each sampling time. Each type of energy reserve was transformed into energetic equivalents using enthalpy combustion (24,000 mJ/mg protein, 17,500 mJ/mg carbohydrates and 39,500 mJ/mg lipids) [13].

### Statistical analysis

Statistical procedures were carried out with STATISTICA [38]. Normality and homogeneity of data were first tested with Shapiro-Wilk and Levene tests. As these assumptions were not met, a non parametric test was applied to analyze the data. Diet effect and time exposure were assessed using Kruskal-Wallis test and Mann-Whitney U test for post-hoc pairwise comparisons. Significance was tested at p < 0.05.

## Results

### Dynamics of the response of digestive enzyme activity levels and energy reserves to food starvation

#### Amylase activity

**Fig 2:**
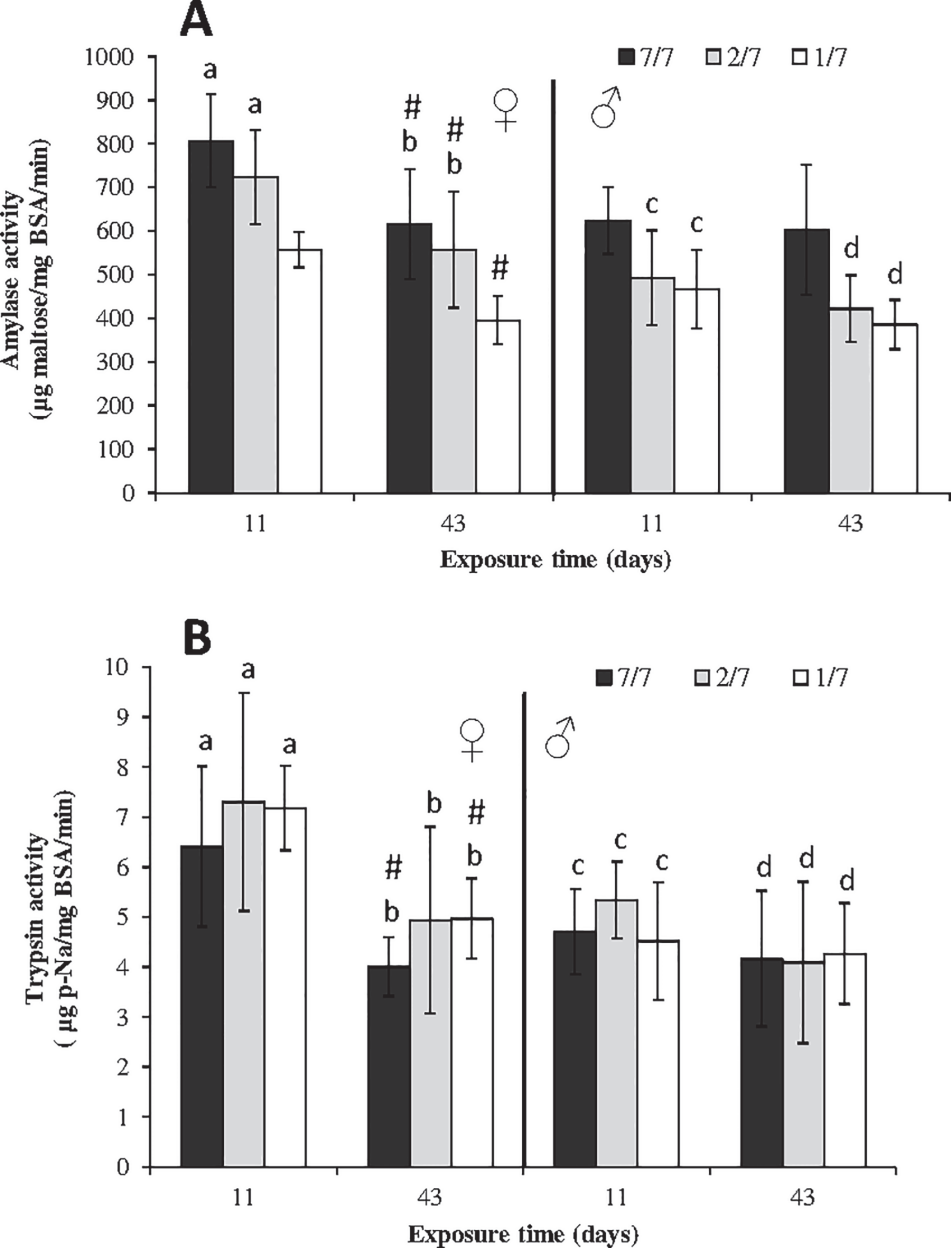
Amylase (A, μg maltose/mg BSA/min) and trypsin (B, μg p-Na/mg BSA/min) activity levels in *Gammarus fossarum* exposed to 3 levels of food starvation (control: fed 7 days a week; 2/7: fed 2 days a week; 1/7: fed 1 day a week) after 11 and 43 days (means ± SD, n = 6). For each date and gender, bars with the same letter were not significantly different (p < 0.05). The hash (#) symbol points to significant changes between the two sampling times for each diet condition (p < 0.05).

For the both cycles, food starvation inhibited significantly amylase activity in males (p = 0.0394 for day 11; p = 0.0084 for day 43) and females (p = 0.0044 for day 11; p = 0.0124 for day 43) (Fig 2A). In females, significant differences between the most severely starved organisms (fed 1/7) and the controls (fed 7/7) were observed. Amylase activity was reduced by 31% and 36%, compared to the controls after 11 (p = 0.0039) and 43 days (p = 0.039) of starvation, respectively (Fig 2A). Between the two sampling times, amylase activity significantly decreased by 24 (p = 0.025), 23 (p = 0.037) and 29% (p = 0.004) for each of the 7/7, 2/7 and 1/7 feeding conditions in females. In males, amylase activity in the two starved conditions (1/7 and 2/7) was significantly lower than in the controls (1/7: p = 0.024 for day 11 and p = 0.0034 for day 43; and 2/7: p = 0.037 for day 11 and p = 0.024 for day 43) (Fig 2A). The highest starvation level (1/7) reduced amylase activity by 25 and 36% after 11 and 43 days, respectively, compared to the control, but no difference was noticed according to the length of food starvation (11 and 43 days) in males.

#### Trypsin activity

Food starvation had no influence on trypsin activity levels whether in males (p = 0.262 for day 11 and p = 0.8054 for day 43) or in females (p = 0.5318 for day 11 and p = 0.2291 for day 43) (Fig 2B). Inversely, trypsin activity significantly decreased in females (Fig 2B) between the first (11 days) and the second (43 days) reproductive cycles (p = 0.0064 and 0.004 for females fed (7/7) and starved (1/7) respectively). For example, between 11 and 43 days, trypsin activity decreased by 37.5% and 31% in control gammarids (7/7), and in the most severely starved females (1/7), respectively. In males, values ranged between 4 (±1.6) and 5.3 (±0.7) μg p-Na/mg BSA/min, whatever the starvation level or exposure length.

#### Energy reserves

**Fig 3:**
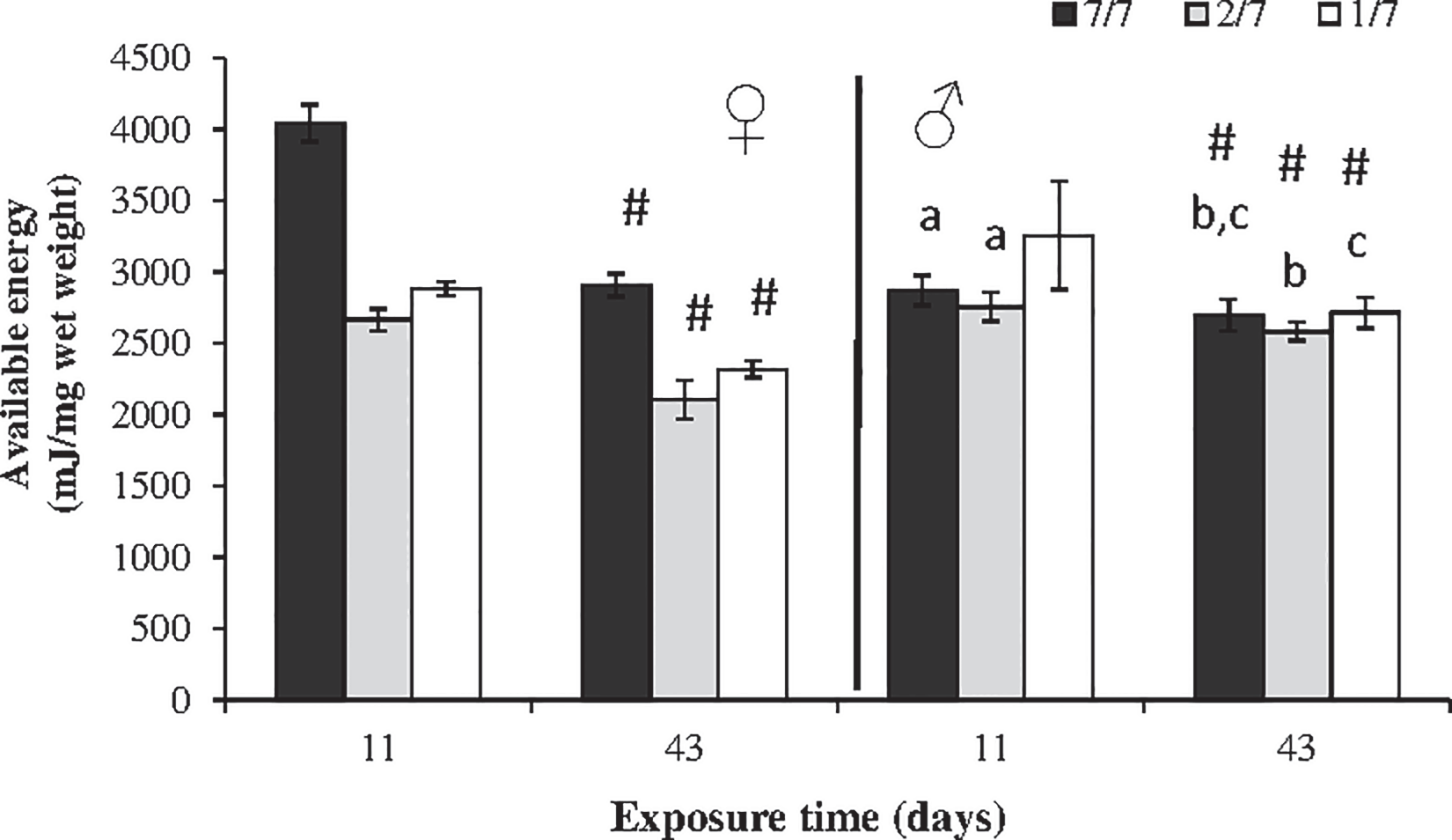
Available energy (mJ/mg wet weight) in *Gammarus fossarum* exposed to 3 levels of food starvation (controls: fed 7 days a week; 2/7: fed 2 days a week; 1/7: fed 1 day a week) after 11 and 43 days (means ± SD, n = 6). For each date and gender, bars with the same letter were not significantly different (p < 0.05). The hash (#) symbol points significant changes between the two sampling times in each diet condition (p < 0.05).

In females, food starvation significantly reduced available energy for the both cycle (p = 0.0005 for day 11 and p = 0.0008 for day 43) (Fig 3). From the first reproductive cycle (after 11 days), available energy values in 1/7 and 2/7 females were about one third lower than in the controls (p = 0.0039 between 1/7 and 7/7 and p = 0.0039 between 2/7 and 7/7). Over the second reproductive cycle (after 43 days), 1/7 and 2/7 females exhibited average energy values 20% and 28% lower than available energy in control females (p = 0.0039 between 1/7 and 7/7 and p = 0.039 between 2/7 and 7/7).

In males, food starvation did not affected available energy. In addition, after 11 days, the most severely starved males (fed one day a week) even exhibited significant higher (p = 0.0064 between 1/7 and 7/7 and p = 0.0.039 between 1/7 and 2/7) level of available energy.

In contrast, in both males and females and for all diets, available energy significantly decreased between the two sampling times (11 and 43 days). In females, the decrease ranged between 563 mJ/mg of wet weight (2/7) and 1,137 mJ/mg of wet weight (controls) (p = 0.004; 0.002 and 0.002 for females (7/7), (2/7) and (1/7) respectively). In males, the energy loss was less conspicuous: it ranged between 171 mJ/mg of wet weight (controls) and 538 mJ/mg of wet weight (1/7) (p = 0.004; 0.0104 and 0.025 for males (7/7), (2/7) and (1/7) respectively).

### Survival, molt stage and reproductive success

Male and female survival rates decreased concurrently with starvation length, with average values of 98% and 86.5% after 11 days and 43 days of starvation, respectively (Table 1). The molting stages of females exposed to starvation are summarized in Table 1. After 11 days of exposure, all females were at molt stage AB or C1 whatever the starving condition. After 43 days, all females fed *ad libitum* had started their second molting cycle, while 4 and 14% of the females fed 2 days and 1 day a week were just finishing their first molting cycle. These results show that only females at the AB or C1 stage were free of the influence of molting on the responses under study.

**Table 1:** Occurrence (%) of different molt stages (AB, C1, C2, D1 and D2) in *Gammarus fossarum* females, and survival rates (%) in males and females exposed to 3 levels of food starvation (control: fed 7 days a week; 2/7: fed 2 days a week; 1/7: fed 1 day a week) after 11 and 43 days.

Time of exposure	Food availability	♀ Molt stages					Survival rates	
(days)	(day/day)	(%)					(%)	
		AB	C1	C2	D1	D2	males	females
11	7/7	40	60	0	0	0	100	97
	2/7	67	33	0	0	0	97	97
	1/7	53	47	0	0	0	97	100
43	7/7	43	57	0	0	0	93	79
	2/7	81	15	0	0	4	89	80
	1/7	73	13	0	0	14	89	89

**Fig 4:**
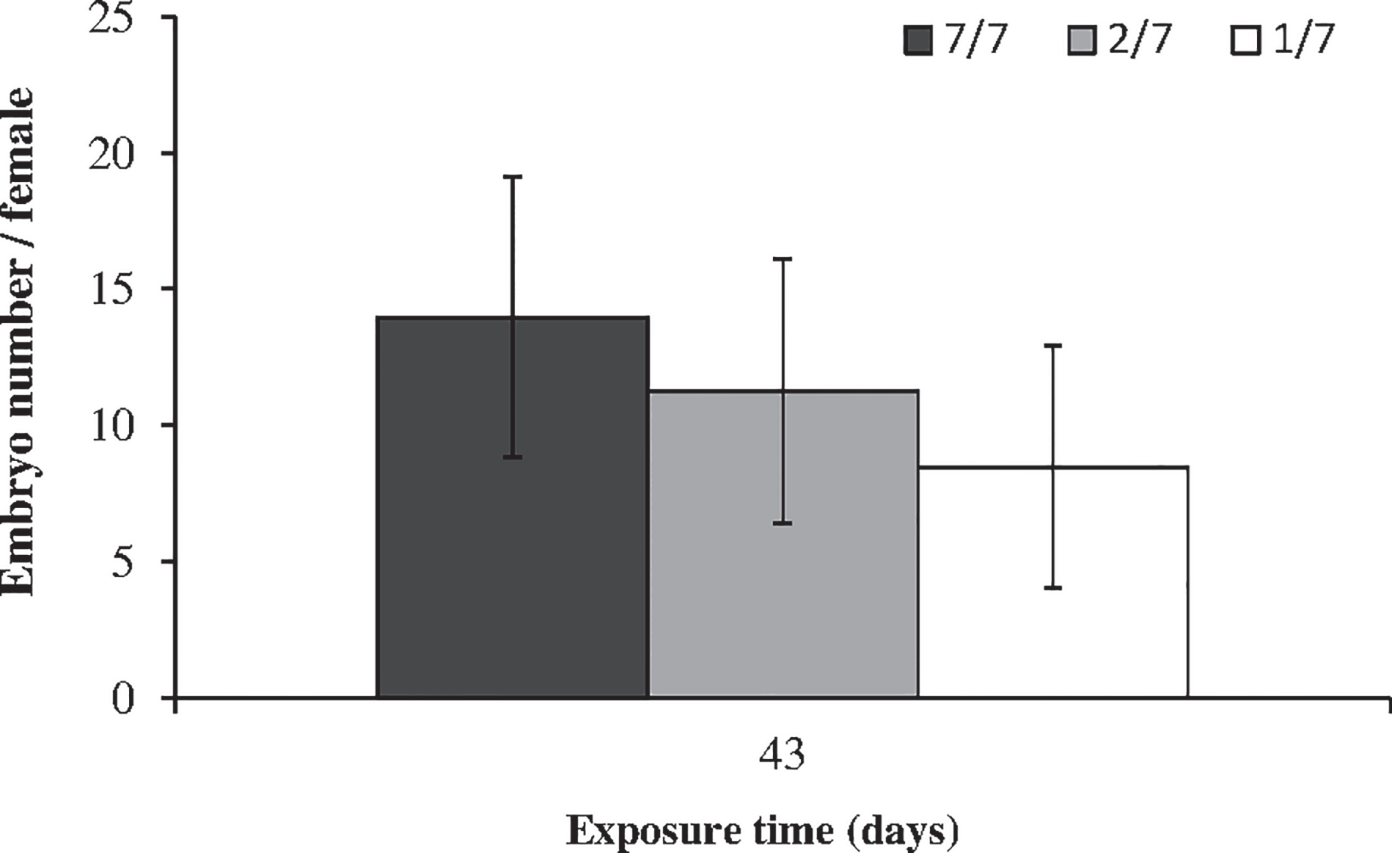
Mean embryo numbers in *Gammarus fossarum* females exposed to 3 levels of diet starvation (control: fed 7 days a week; 2/7: fed 2 days a week; 1/7: fed 1 day a week) after 43 days (means ± SD, n = 6). Bars with the same letter were not significantly different (p < 0.05).

The number of embryos *per* female (*i*.*e*. fertility) was counted after 43 days of food stress (Fig 4). Only embryos from females at the AB or C1 stage were investigated. Starvation resulted in an embryo loss of 19% in 2/7 females (p = 0.025) and 39% in 1/7 females (p = 0.0039), compared to the controls fed 7/7 days.

## Discussion

### Effect of food restriction on energy metabolism and reproductive parameters

This study focuses on energy metabolism impairments related to food starvation through the assessment of its biological effects at the individual level, including the reproductive outcomes. In order to assess the effect of exposure time, gammarids were sampled after 11 and 43 days, when most females were at the beginning of their reproductive cycle for all our diet conditions. This way, for each sex and for each parameter, similar patterns were observed after 11 and 43 days. These results show an early change in amylase activity during the first reproductive cycle in response to lower food intake. A similar pattern was observed concerning energy reserves in females. As regards digestive activity, amylase activity appeared to be downregulated when food was less available, whereas trypsin activity remained unaltered. This difference between amylase and trypsin responses could be linked to the nature of the food, which was solely composed of plant material and contained no animal protein. This effect of food quantity and quality on digestive enzymes in *Gammarus fossarum* is more extensively discussed in a previous study carried out in the same stress conditions [30]. Consequently, lower food availability (1/7 or 2/7 days feed) decreased the digestive capacity and possibly contributed to a lower energy access for starved gammarids. Otherwise, it may be hypothesized that the reduction of digestive processes was a means to decrease energy expenditure during starvation. Food acquisition and assimilation are expensive processes, so decreased digestive enzyme activity can be a strategy for energy saving. This reduction in digestive energy expenditure is part of an overall bodily reduction of energy expenditure during starvation [39]. *G*. *fossarum* can also decrease their energy expenditure after one week of starvation by reducing their oxygen consumption and their ventilatory and locomotor activity [40]. This is in agreement with the findings of Mezek *et al*. [41] and García-Esquivel *et al*. [42], who reported that starvation resulted in a significant decrease in electron transport system (ETS) activity, directly linked to the oxygen consumption process.

Besides, available energy was significantly lower in starved females than in control females fed *ad libitum*. By contrast, no effect of food starvation on available energy was noted in males whatever the diet. These energy responses highlight differences in metabolic needs between males and females. In females, all the energy has to be allocated to maintaining the basal metabolism and ensuring the reproductive cycle. Therefore, food starvation affected available energy in females throughout the whole period of deprivation. Many studies have shown higher lipid contents in female gammarids than in males [37, 43–45]. This influence of sex on reserve composition is usually attributed to the oogenesis process. This process is related to the synthesis of lipoproteins taking place in females over the reproductive cycle. Gammarids reproduce continuously, so in parallel to their energy uptake, their reproduction can be constantly fuelled from stored substrates if enough food is available [46]. Moreover in the current study, feed was exclusively composed of plant material. This may have had a more severe effect on females than on males, as *Echinogammarus marinus* females consume significantly more animal material at the expense of algae [47].

However, similar effects of food starvation were recorded in both males and females over the two reproductive cycles. A significant decrease in digestive activity in starved and control females and a drop in available energy in both males and females was highlighted. These observations support that a diet exclusively composed by alder leaves, though fed *ad libitum*, may not thoroughly supply the nutrients required to maintain gammarids’ level of available energy stable. In fact, many studies have mentioned that freshwater gammarids may have high diet diversity composed of algae, fungi and animals [48–51]. This diversified diet seems to be necessary to meet their nutritional requirements. In the same way, in *Dikerogammarus villosus*, variation in growth rates and lipid quantities might be related to variation in the food regime [52]. The results obtained in the current study could be compared with those obtained by Foucreau *et al*. [53], who worked on *Gammarus pulex* fed on leaves for 12 days. They evidenced lower triglyceride contents in males and females, regardless of the leaf species and of population origin.

The possible consequences of energy-based sub-organismal outcomes on higher levels of biological organization was investigated. Several studies on daphnids exposed to various chemical stressors have already underlined the link between CEA and reproduction criteria (intrinsic rate of natural increase, mean number of young *per* female, mean brood size and net reproductive rate) [10, 13, 54]. In the same way, the SfG values measured in daphnids exposed to Cd or food stress was positively correlated with reproduction [55, 56]. Moreover, in contaminated conditions the reduction in gammarid SfG was generally caused by a decreased feeding rate [57, 58]. Finally, a positive correlation was established between SfG and the reproductive outcome in *Gammarus pulex* under zinc pollution [58]. In the same way, this study underlines a time sequence between a reduction in digestive capacity and energy reserves during oogenesis, both related to food availability and indirectly to the energy uptake on the one hand, and a potential reduction in the number of embryos produced *per* female on the other hand. The sensitivity and the precocity of gammarids’ digestive responses, associated with measurements of available energy in females, can be used to assess effects on the reproductive success and to predict potential changes at the population level.

### Assessing environmental risk in aquatic ecosystems using gammarids’ digestive enzyme activity

After 11 days, the most severely starved gammarids (fed once a week) exhibited amylase activity levels reduced by 25 and 31% compared to control males and females, respectively. This rapid decrease in digestive capacity induced a drop in energy reserves and embryo numbers (28%) in females. These results underline (i) similar sensitivity of males and females concerning their digestive capacity, and (ii) the ecological relevance of digestive markers, because they can be used to anticipate future consequences on reproduction in females, a key biological variable for population dynamics. As previously mentioned, many studies have highlighted that chemical contaminants can also influence food assimilation through the impairment of digestive enzyme activity [9]. In the same way, it may be assumed that all types of stressors (including toxic chemical stressors) that inhibit digestive activity in gammarids could represent a risk for female reproduction and possibly to the whole population. In biomonitoring programs, male gammarids have been proposed in an active approach to avoid the influence of biotic (particularly reproduction) parameters [30], known as confounding factors. In view of our results, inhibition of digestive enzyme activity in males during an active biomonitoring program could be a sign of a potential effect on female reproduction. In a previous *in situ* experiment, amylase activity in male gammarids exposed to different contaminated sites for 15 days dropped by up to 54% compared to gammarids in reference sites [26], corresponding to the inhibition level that induced a negative effect on reproduction in the present study.

However, the potential impact of chemical stressors on gammarids assessed in a caging approach might not reflect the biological responses of native populations submitted to chronic chemical stress. Indigenous populations quite probably develop mechanisms of resistance and adaptation to cope with the effects of toxic stress. Complementary studies about inter-population variability should be implemented to further clarify this issue for ecological risk assessment.

## Supporting Information

S1 Table Raw data. Data obtained in male and female gammarids exposed to 3 levels of food starvation (controls: fed 7 days a week; 2/7: fed 2 days a week; 1/7: fed 1 day a week) after 11 or 43 days (n = 6).
(PDF)

## References

[1] Depledge M, Fossi M. The role of biomarkers in environmental assessment (2). Invertebrates. Ecotoxicology. 1994; 3: 161–172. 10.1007/BF00117081 24202002

[2] Handy R, Depledge M. Physiological responses: their measurement and use as environmental biomarkers in ecotoxicology. Ecotoxicology. 1999; 8: 329–349.

[3] Duquesne S. Effects of an organophosphate on *Daphnia magna* at suborganismal and organismal levels: Implications for population dynamics. Ecotox Environ Saf. 2006; 65: 145–150. 16545452 10.1016/j.ecoenv.2006.01.008

[4] Forbes VE, Palmqvist A, Bach L. The use and misuse of biomarkers in ecotoxicology. Environ Tox Chem. 2006; 25: 272–280. 10.1897/05-257r.1 16494252

[5] Xuereb B, Lefèvre E, Garric J, Geffard O. Acetylcholinesterase activity in *Gammarus fossarum* (Crustacea Amphipoda): Linking AChE inhibition and behavioural alteration. Aquatic Tox. 2009; 94: 114–122. 10.1016/j.aquatox.2009.06.010 19608286

[6] De Coen W, Janssen C, Giesy J. Biomarker applications in ecotoxicology: bridging the gap between toxicology and ecology. In: Persoone G, Colin J, De Coen W, editors. New microbiotests for routine toxicity screening and biomonitoring, 2000. pp. 13–25.

[7] Lacaze E, Geffard O, Goyet D, Bony S, Devaux A. Linking genotoxic responses in *Gammarus fossarum* germ cells with reproduction impairment, using the Comet assay. Environ Res. 2011; 111: 626–634. 10.1016/j.envres.2011.03.012 21489518

[8] De Coen WM, Janssen CR. The use of biomarkers in *Daphnia magna* toxicity testing. I. The digestive physiology of daphnids exposed to toxic stress. Hydrobiologia, 1998; 367: 199–209.

[9] Amiard-Triquet C, Amiard JC, Rainbow PS. Ecological Biomarkers: Indicators of Ecotoxicological Effects. New York: CRC Press; 2013.

[10] De Coen WM, Janssen CR. The missing biomarker link: Relationships between effects on the cellular energy allocation biomarker of toxicant-stressed *Daphnia magna* and corresponding population characteristics. Environ Tox Chem. 2003; 22: 1632–1641. 12836990

[11] Olsen GH, Carroll J, Sva E, Camus L. Cellular energy allocation in the Arctic sea ice amphipod *Gammarus wilkitzkii* exposed to the water soluble fractions of oil. Mar Environ Res. 2008; 66: 213–214. 10.1016/j.marenvres.2008.02.063 18381222

[12] Dutra B, Fernandes F, Lauffer A, Oliveira G. Carbofuran-induced alterations in the energy metabolism and reproductive behaviors of *Hyalella castroi* (Crustacea, Amphipoda). Comp Biochem Physiol, Part C: Toxicol Pharmacol. 2009; 149: 640–646. 19358339 10.1016/j.cbpc.2009.01.005

[13] De Coen WM, Janssen CR. The use of biomarkers in *Daphnia magna* toxicity testing. IV. Cellular Energy Allocation: a new methodology to assess the energy budget of toxicant-stressed Daphnia populations. J Aquat Ecosyst Stress Recov. 1997; 6: 43–55.

[14] Naylor C, Pindar L, Calow P. Inter- and intraspecific variation in sensitivity to toxins; the effects of acidity and zinc on the freshwater crustaceans *Asellus Aquaticus* (L.) and Gammarus pulex (L.). Wat Res. 1990; 24: 757–762.

[15] Kalman J, Palais F, Amiard J, Mouneyrac C, Muntz A, Blasco J, et al Assessment of the health status of populations of the ragworm *Nereis diversicolor* using biomarkers at different levels of biological organisation. Mar Ecol Prog Ser. 2009; 393: 55–67.

[16] Bourgeault A, Gourlay‐Francé C, Vincent‐Hubert F, Palais F, Geffard A, Biagianti‐Risbourg S, et al Lessons from a transplantation of zebra mussels into a small urban river: an integrated ecotoxicological assessment. Environ Toxicol. 2010; 25: 468–478. 10.1002/tox.20591 20549621

[17] Boldina-Cosqueric I, Amiard J-C, Amiard-Triquet C, Dedourge-Geffard O, Métais I, Mouneyrac C, et al Biochemical, physiological and behavioural markers in the endobenthic bivalve *Scrobicularia plana* as tools for the assessment of estuarine sediment quality. Ecotox Environ Saf. 2010; 73: 1733–1741. 10.1016/j.ecoenv.2010.08.008 20797788

[18] Fossi Tankoua O, Buffet P, Amiard J, Amiard-Triquet C, Méléder V, Gillet P, et al Intersite variations of a battery of biomarkers at different levels of biological organisation in the estuarine endobenthic worm *Nereis diversicolor* (Polychaeta, Nereididae). Aquatic Tox. 2012; 114: 96–103. 10.1016/j.aquatox.2012.02.016 22417766

[19] Coulaud R, Geffard O, Xuereb B, Lacaze E, Quéaau H, Garric J, et al In situ feeding assay with *Gammarus fossarum* (Crustacea): Modelling the influence of confounding factors to improve water quality biomonitoring. Wat Res. 2011; 45: 6417–6429. 10.1016/j.watres.2011.09.035 22014562

[20] Dedourge-Geffard O, Charron L, Hofbauer C, Gaillet V, Palais F, Lacaze E, et al Temporal patterns of digestive enzyme activities and feeding rate in gammarids *Gammarus fossarum* exposed to inland polluted waters. Ecotox Environ Saf. 2013; 97: 139–146. 10.1016/j.ecoenv.2013.07.016 23932430

[21] Maltby L, Naylor C. Preliminary observations on the ecological relevance of the Gammarus ‘scope for growth’ assay: effect of zinc on reproduction. Func Ecol. 1990; 4: 393–397.

[22] Kelly DW, Dick JT, Montgomery WI. The functional role of *Gammarus* (Crustacea, Amphipoda): shredders, predators, or both? Hydrobiologia. 2002; 485: 199–203.

[23] Macneil C, Dick JTA, Elwood RW. The dynamics of predation on *Gammarus* spp. (Crustacea: Amphipoda). Biological Rev. 1999; 74: 375–395.

[24] Maltby L. Stress, shredders and streams: using Gammarus energetics to assess water quality In: Sutcliffe DW, editors. Water quality & stress indicators in marine and freshwater systems: linking levels of organisation. Freshwater Biological Association, 1994 pp. 98–110.

[25] Geffard O, Xuereb B, Chaumot A, Geffard A, Biagianti S, Noël C, et al Ovarian cycle and embryonic development in *Gammarus fossarum*: Application for reproductive toxicity assessment. Environ Toxicol Chem. 2010; 29: 2249–2259. 10.1002/etc.268 20872689

[26] Charron L, Geffard O, Chaumot A, Coulaud R, Queau H, Geffard A, et al Effect of water quality and confounding factors on digestive enzyme activities in *Gammarus fossarum* . Environ Sci Pollut Res. 2013; 20: 9044–9056. 10.1007/s11356-013-1921-5 23784059

[27] Pöckl M. Effects of temperature, age and body size on moulting and growth in the freshwater amphipods *Gammarus fossarum* and *G*. *roeseli* . Freshwater Biol. 1992; 27: 211–225.

[28] Sutcliffe DW. Reproduction in *Gammarus* (Crustacea, Amphipoda): basic processes. Freshwater Forum. 2010; 2:102–128.

[29] Maltby L, Clayton SA, Wood RM, Mcloughlin N. Evaluation of the *Gammarus pulex in situ* feeding assay as a biomonitor of water quality: Robustness, responsiveness, and relevance. Environ Toxicol Chem. 2002; 21: 361–368. 11833806

[30] Charron L, Geffard O, Chaumot A, Coulaud R, Jaffal A, Gaillet V, et al Influence of molting and starvation on digestive enzyme activities and energy storage in Gammarus fossarum. Plos One. 2014; 9: e96393 10.1371/journal.pone.0096393 24788197 PMC4005779

[31] Besse JP, Coquery M, Lopes C, Chaumot A, Budzinski H, Labadie P, et al Caged *Gammarus fossarum* (crustacea) as a robust tool for the characterization of bioavailable contamination levels in continental waters. Toward the determination of threshold values. Wat Res. 2013; 47: 650–660. 10.1016/j.watres.2012.10.024 23182666

[32] Palais F, Jubeaux G, Dedourge-Geffard O, Giambérini L, Biagianti-Risbourg S, Geffard A. Amylolytic and cellulolytic activities in the cristalline style and the digestive diverticulae of the freshwater bivalve *Dreissena polymorpha* (Pallas,1771). Mollusc Res. 2010; 30: 29–36.

[33] Garcia-Carreño FL, Haard NF. Characterization of proteinase classes in langostilla (*Pleuroncodes planipes*) and crayfish (*Pacifastacus Planipes*) extracts. J of Food Biochem. 1993; 17: 97–113.

[34] Bradford MM. A rapid and sensitive method for the quantitation of microgram quantities of protein utilizing the principle of protein-dye binding. Anal Biochem. 1976; 72: 248–254. 942051 10.1016/0003-2697(76)90527-3

[35] Van Handel E. Rapid determination of glycogen and sugars in mosquitoes. J of the American Mosquito Control Association. 1985a; 1: 299–301. 2906671

[36] Van Handel E. Rapid determination of total lipids in mosquitoes. J of the American Mosquito Control Association. 1985b; 1: 302–304. 2906672

[37] Plaistow SJ, Bollache L, Cézilly F. Energetically costly precopulatory mate guarding in the amphipod *Gammarus pulex*: causes and consequences. Animal Behaviour. 2003; 65: 683–691. 12566098

[38] StatSoft, Inc. STATISTICA, TULSA, Oklahoma USA.

[39] Wang T, Hung CC, Randall DJ. The comparative physiology of food deprivation: from feast to famine. Annual Review of Physiology. 2006; 68: 223–251. 16460272 10.1146/annurev.physiol.68.040104.105739

[40] Hervant F, Mathieu J, Barré H, Simon K, Pinon C. Comparative study on the behavioral, ventilatory, and respiratory responses of hypogean and epigean crustaceans to long-term starvation and subsequent feeding. Comp Biochem Physiol Part A Physiol. 1997; 118: 1277–1283.

[41] Mezek T, Simčič T, Arts MT, Brancelj A. Effect of fasting on hypogean (Niphargus stygius) and epigean (*Gammarus fossarum*) amphipods: a laboratory study. Aquat Ecol. 2010; 44: 397–408.

[42] García-Esquivel Z, Bricelj VM, Felbeck H. Metabolic depression and whole-body response to enforced starvation by *Crassostrea gigas* postlarvae. Comp Biochem Physiol Part A Physiol. 2002; 133: 63–77. 12160873 10.1016/s1095-6433(02)00112-5

[43] Meier G, Meyer E, Meyns S. Lipid content of stream macroinvertebrates. Archiv für Hydrobiologie. 2000; 147: 447–463. 10.1055/s-0033-1360260 24715412

[44] Sroda S, Cossu-Leguille C. Seasonal variability of antioxidant biomarkers and energy reserves in the freshwater gammarid *Gammarus roeseli* . Chemosphere. 2011; 83: 538–544. 10.1016/j.chemosphere.2010.12.023 21215985

[45] Gismondi E, Beisel JN, Cossu-Leguille C. Influence of gender and season on reduced glutathione concentration and energy reserves of *Gammarus roeseli* . Environ Res. 2012; 160: 17–23. 10.1016/j.envpol.2011.09.021 22769238

[46] Koop JH, Schäffer M, Ortmann C, Winkelmann C. Towards environmental assessment of river ecosystems by analyzing energy reserves of aquatic invertebrates. Limnologica-Ecology and Management of Inland Waters. 2008; 38: 378–387.

[47] Dick JT, Johnson MP, Mccambridge S, Johnson J, Carson VE, Kelly DW, et al Predatory nature of the littoral amphipod *Echinogammarus marinus*: gut content analysis and effects of alternative food and substrate heterogeneity. Mar Ecol. Prog Ser. 2005; 291: 151–158.

[48] Bärlocher F, Kendrick B. Fungi in the diet of *Gammarus pseudolimnaeus* (Amphipoda). Oikos. 1973; 24: 295–300.

[49] Willoughby L, Sutcliffe D. Experiments on feeding and growth of the amphipod *Gammarus pulex* (L.) related to its distribution in the River Duddon. Freshwater Biology. 1976; 6: 577–586.

[50] Macneil C, Dick JTA, Elwood RW. The Trophic ecology of freshwater *Gammarus* spp. (crustacea: amphipoda): problems and perspectives concerning the fonctional feeding group concept. Biological Review. 1997; 72: 349–364.

[51] Felten V, Tixier G, Guérold F, De Crespin DB, Dangles O. Quantification of diet variability in a stream amphipod: implications for ecosystem functioning. Fundamental and Applied Limnology. 2008; 170: 303–313.

[52] Maazouzi C, Piscart C, Pihan JC, Masson G. Effect of habitat-related resources on fatty acid composition and body weight of the invasive *Dikerogammarus villosus* in an artificial reservoir. Fundamental and Applied Limnology. 2009; 175: 327–338.

[53] Foucreau N, Piscart C, Puijalon S, Hervant F. Effect of Climate-Related Change in Vegetation on Leaf Litter Consumption and Energy Storage by *Gammarus pulex* from Continental or Mediterranean Populations. Plos One. 2013; 8: e77242 10.1371/journal.pone.0077242 24204778 PMC3799701

[54] Muyssen BT, Janssen CR, Bossuyt BT. Tolerance and acclimation to zinc of field-collected *Daphnia magna* populations. Aquat Toxicol. 2002; 56: 69–79. 11755696 10.1016/s0166-445x(01)00206-5

[55] Baillieul M, Selens M, Blust R. Scope for growth and fitness of *Daphnia magna* in salinity-stressed conditions. Func Ecol. 1996; 10: 227–233.

[56] Baillieul M, Smolders R, Blust R. The effect of environmental stress on absolute and mass-specific scope for growth in *Daphnia magna* Strauss. Comp Biochem Physiol, Part C: Toxicol Pharmacol. 2005; 140: 364–373. 15893503 10.1016/j.cca.2005.03.007

[57] Naylor C, Maltby L, Calow P. Scope for growth in Gammarus pulex, a freshwater benthic detritivore. Hydrobiologia. 1989; 188/189: 517–523.

[58] Maltby L, Naylor C, Calow P. Field deployment of a scope for growth assay involving *Gammarus pulex*, a freshwater benthic invertebrate. Ecotox Environl Saf. 1990 19: 292–300. 2364912 10.1016/0147-6513(90)90031-y

