# Supplementary material for: Consequences of Lower Food Intake on the Digestive Enzymes Activities, the Energy Reserves and the Reproductive Outcome in Gammarus fossarum
Source: PLoS One. 2015 Apr 16;10(4):e0125154. doi: 10.1371/journal.pone.0125154 (PMC4400123; doi:10.1371/journal.pone.0125154)
Supplement: S1 Table — Data obtained in male and female gammarids exposed to 3 levels of food starvation (controls: fed 7 days a week; 2/7: fed 2 days a week; 1/7: fed 1 day a week) after 11 or 43 days (n = 6). (PDF) [file pone.0125154.s001.pdf]

Sex	Exposure time (days)	Level of food starvation	Repetition	Amylase (µg maltose/mg BSA/min)	Trypsin (µg p-Na/mg BSA/min)	Available energy (mJ/mg)	Embryo numbers
Female	11	1/7	1	581,3210461	7,801120995	2813,938364	
Female	11	1/7	2	620,5926085	6,999300515	2952,318852	
Female	11	1/7	3	517,5429663	8,297405257	2929,37251	
Female	11	1/7	4	569,3682662	7,500414184	2869,431047	
Female	11	1/7	5	516,9823112	6,266782386	2881,37251	
Female	11	1/7	6	537,9569711	6,199429776	2868,962754	
Female	11	2/7	1	851,9138003	7,701588905	2582,429464	
Female	11	2/7	2	728,1564105	9,945415217	2564,868489	
Female	11	2/7	3	636,2531536	4,913775847	2729,473367	
Female	11	2/7	4	789,1884014	9,559541852	2749,844098	
Female	11	2/7	5	777,035421	6,745046536	2672,575806	
Female	11	2/7	6	560,2881168	4,938219785	2700,907513	
Female	11	7/7	1	827,0872739	6,707757759	4134,401533	
Female	11	7/7	2	869,4966288	6,394621998	4090,499674	
Female	11	7/7	3	657,1574065	6,366821966	4131,006991	
Female	11	7/7	4	902,1551235	9,238642134	3817,250894	
Female	11	7/7	5	689,8478905	4,636723941	3961,48504	
Female	11	7/7	6	893,7876001	5,120557625	4125,855772	
Female	43	1/7	1	368,4376191	4,29509054	2286,805638	7
Female	43	1/7	2	423,6730458	6,029667079	2279,547102	10
Female	43	1/7	3	413,2912927	5,509685953	2259,410516	6
Female	43	1/7	4	400,6251513	4,263673963	2417,92759	8
Female	43	1/7	5	464,2524632	5,521230352	2350,259297	9
Female	43	1/7	6	302,4224282	4,224256778	2316,542224	11
Female	43	2/7	1	523,0552023	6,851099428	2055,496708	12
Female	43	2/7	2	609,0403916	5,267815532	2045,428415	11
Female	43	2/7	3	307,9186904	2,737095767	1906,813781	10
Female	43	2/7	4	596,6015482	2,797279852	2283,555244	14
Female	43	2/7	5	621,8349021	5,00005456	2103,965001	10

Female	43	2/7	6	684,2333877	6,985726558	2227,674524	11
Female	43	7/7	1	559,4040565	4,790930548	2764,130236	13
Female	43	7/7	2	778,6802946	3,376194596	2924,754626	13
Female	43	7/7	3	492,7730647	4,390053967	2961,485753	13
Female	43	7/7	4	720,5028802	3,911900799	2954,022919	13
Female	43	7/7	5	527,9697776	3,542066867	2928,96926	15
Female	43	7/7	6	614,7543674	4,056754592	2906,526898	17
Male	11	1/7	1	480,9698435	5,242010865	2950,445727	
Male	11	1/7	2	523,7095554	4,710360366	3029,32877	
Male	11	1/7	3	378,3824588	6,341537377	3121,13841	
Male	11	1/7	4	335,205693	3,146180694	3238,91402	
Male	11	1/7	5	523,8179442	3,53035044	3999,187191	
Male	11	1/7	6	557,5142389	4,133200683	3187,635971	
Male	11	2/7	1	396,5741271	5,06416766	2844,117267	
Male	11	2/7	2	646,7581969	4,989114272	2813,444097	
Male	11	2/7	3	606,3214574	6,462852122	2752,097755	
Male	11	2/7	4	393,6672401	5,673849292	2579,766048	
Male	11	2/7	5	436,9459884	5,635461856	2841,541658	
Male	11	2/7	6	478,3799402	4,216114056	2709,014828	
Male	11	7/7	1	660,6424544	4,33346013	2734,684212	
Male	11	7/7	2	477,3365962	4,520762696	2766,969927	
Male	11	7/7	3	675,6783218	6,162859705	2843,255641	
Male	11	7/7	4	615,7664395	5,24571446	2932,969927	
Male	11	7/7	5	632,7557861	3,829694291	2949,82707	
Male	11	7/7	6	681,8762336	4,165820078	2998,969927	
Male	43	1/7	1	356,7485443	5,640187177	2581,074136	
Male	43	1/7	2	456,6432877	3,126759071	2868,371697	
Male	43	1/7	3	406,5435142	4,913325809	2644,527794	
Male	43	1/7	4	341,8104217	4,695458511	2817,327794	
Male	43	1/7	5	438,4534572	4,117374935	2701,425355	
Male	43	1/7	6	313,8220979	3,119526157	2685,269258	

Male	43	2/7	1	425,8985638	3,760173533	2563,063872	
Male	43	2/7	2	490,464589	3,633177669	2560,492444	
Male	43	2/7	3	507,8104966	3,438000921	2505,635301	
Male	43	2/7	4	313,1843085	3,813354287	2640,880481	
Male	43	2/7	5	353,2305314	2,647598326	2678,778158	
Male	43	2/7	6	440,763702	7,276567542	2550,492444	
Male	43	7/7	1	489,8719415	4,127035039	2718,371504	
Male	43	7/7	2	870,1056033	6,213152414	2722,085789	
Male	43	7/7	3	625,1521848	3,768104907	2484,267555	
Male	43	7/7	4	471,3219667	5,129621543	2778,371504	
Male	43	7/7	5	519,3615582	3,453805794	2714,371504	
Male	43	7/7	6	641,1847136	2,304431223	2788,085789	
